# Optimization of Indocyanine Green for Intraoperative Fluorescent Image-Guided Localization of Lung Cancer; Analysis Based on Solid Component of Lung Nodule

**DOI:** 10.3390/cancers15143643

**Published:** 2023-07-16

**Authors:** Ok Hwa Jeon, Byeong Hyeon Choi, Jiyun Rho, Kyungsu Kim, Jun Hee Lee, Jinhwan Lee, Beop-Min Kim, Hyun Koo Kim

**Affiliations:** 1 Department of Thoracic and Cardiovascular Surgery, Korea University Guro Hospital, Korea University College of Medicine, Seoul 08308, Republic of Korea; hwa1983418@gmail.com (O.H.J.); baby2music@gmail.com (B.H.C.); jiyun.r1219@gmail.com (J.R.); k_gangter@korea.ac.kr (K.K.); lee2632@naver.com (J.H.L.); 2 Department of Biomedical Sciences, Korea University College of Medicine, Seoul 02841, Republic of Korea; 3 Department of Pathology, Myongji Hospital, Goyang 10475, Republic of Korea; jinhwan8734@gmail.com; 4 Department of Biomedical Engineering, Korea University College of Health Science, Seoul 02841, Republic of Korea; bmk515@korea.ac.kr

**Keywords:** intraoperative fluorescent image, localization of lung cancer, solid component of lung nodule

## Abstract

**Simple Summary:**

Multiple studies have performed intravenous injections of indocyanine green (ICG) for lung cancer detection, including primary and metastatic lung cancers. However, there is no consensus regarding the optimization of the ICG injection method. We optimized the ICG injection method (2 mg/kg at 12 h before surgery) using a rabbit model of lung cancer for the first time. Additionally, ICG-based cancer identifications are limited to solid tumors, and no study has examined lung ground-glass nodules (GGNs). We investigated the feasibility of ICG-based lung cancer imaging in adenocarcinoma presenting with GGNs and a consolidation-to-tumor (C/T) ratio ≤ 50% for the first time. Overall, 51 cases of lung cancer with a C/T ratio > 50% were successfully revealed with a 95% detection rate. Therefore, this study may provide guidance regarding ICG use for lung cancer detection, especially early-stage lung cancer.

**Abstract:**

ICG fluorescence imaging has been used to detect lung cancer; however, there is no consensus regarding the optimization of the indocyanine green (ICG) injection method. The aim of this study was to determine the optimal dose and timing of ICG for lung cancer detection using animal models and to evaluate the feasibility of ICG fluorescence in lung cancer patients. In a preclinical study, twenty C57BL/6 mice with footpad cancer and thirty-three rabbits with VX2 lung cancer were used. These animals received an intravenous injection of ICG at 0.5, 1, 2, or 5 mg/kg, and the cancers were detected using a fluorescent imaging system after 3, 6, 12, and 24 h. In a clinical study, fifty-one patients diagnosed with lung cancer and scheduled to undergo surgery were included. Fluorescent images of lung cancer were obtained, and the fluorescent signal was quantified. Based on a preclinical study, the optimal injection method for lung cancer detection was 2 mg/kg ICG 12 h before surgery. Among the 51 patients, ICG successfully detected 37 of 39 cases with a consolidation-to-tumor (C/T) ratio of >50% (TNR: 3.3 ± 1.2), while it failed in 12 cases with a C/T ratio ≤ 50% and 2 cases with anthracosis. ICG injection at 2 mg/kg, 12 h before surgery was optimal for lung cancer detection. Lung cancers with the C/T ratio > 50% were successfully detected using ICG with a detection rate of 95%, but not with the C/T ratio ≤ 50%. Therefore, further research is needed to develop fluorescent agents targeting lung cancer.

## 1. Introduction

Lung cancer is the leading cause of cancer-related deaths worldwide [1]. Recently, with the implementation of low-dose computed tomography (CT) in high-risk groups, the diagnosis of small-size early lung cancer, especially lung cancer with ground-glass nodules (GGN), has increased significantly [2]. For patients with small-sized early-stage lung cancer, surgery is the best option to ensure long-term survival. It can increase the 5-year survival rate ten-fold compared to patients who do not undergo surgery [3]. The standard surgical method is lobectomy or more extensive resection of lung tissue to complete the resection, even for small-sized cancers [4,5]. Recently, according to the JCOG 0802 results, there was no significant difference in all parameters, including intra and postoperative complications, between segmentectomy and lobectomy for patients with small lung cancers ≤ 2 cm in diameter and with a consolidation-to-tumor (C/T) ratio > 50% [6]. The 5-year disease-free survival rate after surgery for lung adenocarcinoma in situ and minimally invasive adenocarcinoma is 100% [7]. Therefore, considering that the median age at lung cancer diagnosis is 70 years, which is usually accompanied by extensive comorbidities and poor lung function [8], limited resection, such as segmentectomy or wedge resection, should be performed for these patients with small-sized early-stage lung cancers [5,6,9,10].

Video-assisted thoracoscopic surgery (VATS) and robotic surgery are minimally invasive surgical techniques that are commonly used in lung cancer resection [11,12,13,14]. However, the use of these surgeries to identify lung nodules is limited because of the inability to visually examine the lesion or to directly contact the lesion during surgery [15]. Researchers have developed several methods for preoperative positioning of pulmonary nodules, such as the use of hook wires, micro-coils [16,17], radiotracers, radioactivity contrasts, and dyes to localize cancer lesions during minimally invasive surgery [18,19]. These not only lead to postoperative complications, such as pneumothorax and hemorrhage, but also have a high probability of radiation exposure. Moreover, because this technique can only perform tumor localization, it is difficult to accurately identify the margin of resection.

In 2009, intravenously injected indocyanine green (ICG) was first used for intraoperative observation of hepatocellular carcinoma [20], and this method was subsequently used for the detection of many types of cancer [21,22,23,24,25]. Lung cancer detection using ICG was also conducted, most of which involved the injection of ICG at 5 mg/kg 24 h before surgery [26,27,28,29]. The selected doses and timepoints were based on preclinical studies (i.e., a mouse flank tumor model and canine abdominal wall sarcoma) [27,30]. However, in our previous study, we demonstrated that even a low dose of ICG (1 mg/kg) can be used in the cases of pulmonary neoplasms [31]. Moreover, our previous study confirmed that 1 mg/kg and 2 mg/kg at 3 h and 12 h before surgery, respectively, are the optimal injection conditions for ICG detection in thymoma and esophageal cancer, respectively [23,24].

Furthermore, in the lung supplied by different parts of the circulatory system, we assumed that intravenously injected ICG shows a different distribution in the lung tissue. Therefore, the exact optimal dosage and injection time of ICG for detecting lung cancer remains to be defined. Previous studies using ICG to detect lung cancer were lacking in their inclusion of GGN because of the limited number of patients recruited. Thus, the goal of our study was to identify the optimal time and dose of ICG injection for the intraoperative imaging of lung cancer. The feasibility of using the optimized ICG injection method to detect lung cancer and lung GGNs was also evaluated in patients with lung cancer.

## 2. Materials and Methods

### 2.1. Animal Experiment

All study procedures, including animal care and handling, were approved by the Institutional Animal Care and Use Committee of Korea University (KOREA-2016-0246). To assist the animals in adapting to their environment, 5 mice or rabbits were housed in individual cages with freely available food and water supplies for 1–2 weeks, in accordance with our humane animal care protocols.

### 2.2. Footpad Tumor Detection by Intravenous Injection of Indocyanine Green in Mouse Model

Lewis lung carcinoma–green fluorescent protein (LLC-GFP, Creative Biogene, Shirley, NY, USA) cell lines were used to establish mouse models of footpad cancer. Twenty 6-week-old female C57BL/6 mice were selected and used for tumor implantation. LLC cells (2 × 10^5^ cells/mL) were injected into the footpad of the mice, and tumor volumes were measured using the formula: Volume = ½ (L × W^2^) [32].

The distribution of ICG (Daiichi-Sankyo, Seoul, Republic of Korea) was assessed after 0.5, 1, 2, and 5 mg/kg ICG administration (3, 6, 12, and 24 h) in a mouse model of footpad cancer using an in vivo near-infrared (NIR) imaging system (Davinch Invivo Imaging, Seoul, Republic of Korea). The NIR fluorescence signal was quantified using the ImageJ software (NIH, MD, USA). The tumor-to-normal ratio (TNR) was calculated as the ratio of tumor-to-normal fluorescent signals. [33] To quantify the degree of surgical identification of the tumor by the surgeon, two detectors subjectively evaluated the images of different TNRs. From the quantification, the tumor region with an average TNR greater than 2.0 can be visually observed during surgery.

### 2.3. Lung Cancer Detection by Intravenous Injection of Indocyanine Green in Rabbit Lung Cancer Model

The VX2 rabbit lung cancer model was described in our previous study [34]. To assess tumor formation, three randomly selected rabbits underwent ^18^F-fluorodeoxyglucose positron emission tomography (^18^FDG-PET) using a Gemini TF 16-slice PET scanner. The 30 rabbit models with lung cancer were divided into three groups (1, 2, and 5 mg/kg of ICG). The rabbits were anesthetized using tiletamine–zolazepam (Zoletil 50; Virbac Republic of Korea Inc., Seoul, Republic of Korea) and 5 mg/kg xylazine (RompunTM; Bayer Republic of Korea Inc., Seoul, Republic of Korea) and administered ICG via an ear vein injection. After intubation via tracheostomy, a mechanical ventilator was connected, and anesthesia was maintained with 2% isoflurane (Baxter Healthcare, Deerfield, IL, USA). The unilateral chest wall of rabbit was resected to obtain an image of the entire lung. The distribution of ICG was assessed after 3, 6, 12, and 24 h using an intraoperative color and fluorescence-merged imaging system (ICFIS) and TNR was quantified [23,33]. We also performed lung cancer resection using an endoscopic system in rabbit model. The distribution of ICG in the rabbit lung was visualized in real time using ICFIS, and the lung tumor was surgically removed using endoscopic system.

### 2.4. Human Lung Cancer Study Design

This clinical study was approved by Korea University Guro Hospital (IRB No. 2017GR0075). In total, 51 patients who were preoperatively diagnosed with lung cancer and scheduled to undergo curative surgery were enrolled between June 2015 and July 2017. All patients provided informed consent. Preoperative Chest CT and ^18^FDG-PET/CT was performed for all patients. Patient demographics and intraoperative and postoperative data were also collected. This study excluded patients with liver dysfunction, hypersensitivity, or adverse reactions to ICG, and those receiving neoadjuvant chemotherapy. ICG (2 mg/kg) was intravenously injected into lung cancer patients 12 h before surgery.

### 2.5. Surgical Procedures of Patient with Lung Cancer

Surgical approaches for all patients were discussed individually based on cancer characteristics (location and size) or the surgeon’s preferences. For lung cancers that were peripherally located, with a solid part of < 2 cm, with >50% GGN, and no lymph node metastasis confirmed by preoperative CT, a minimal resection such as a wedge resection or segmentectomy was performed. A lobectomy or pneumonectomy was performed for tumors > 2 cm. All patients underwent surgery through a minimally invasive approach and opted for either VATS or robot-assisted thoracoscopic surgery. Immediately after resection, all the retrieved surgical specimens were investigated ex vivo by means of ICFIS. For tumors with a depth of >0.5 cm, the fluorescence signal was detected after incision to expose the cancer.

We evaluated the feasibility of an ICG injection protocol for lung cancer detection in 11 of the 51 patients using the fluorescence imaging system in our hospital. The NIR fluorescence signal of the lung cancer was evaluated using a Firefly camera PinPoint (da Vinci West, Intuitive Surgical, Sunnyvale, CA USA) or a robotic-assisted system (da Vinci West, CA, USA) in vivo before starting the dissection and ex vivo after resection.

All the specimens were subjected to histological examination. Pathological staging of lung cancer was based on the World Health Organization (WHO) histological classification and the Union for International Union Cancer Control (UICC, eighth edition) TNM classification.

### 2.6. Statistical Analyses

Descriptive data were expressed as means and standard deviations. A two-way ANOVA test was used to determine the effect of injection time and dose of ICG on TNR in mice and rabbit model. The TNR of mice and rabbits at each time point injected at each different dose was compared by one-way ANOVA. One-way ANOVA was used to evaluate the differences in TNR between the lung cancer types, histological type, TNM stage. A t-test was used to evaluate the differences in TNR between the lung cancer and C/T ratio. Correlation analysis was used to evaluate between TNR and tumor size. Statistical significance was set at *p* < 0.05. Statistical analysis was performed using IBS SPSS Statistics 22.0 (IBM Corp., Armonk, NY, USA) in consultation with the Medical Statistical Consulting Center of Korea University Guro Hospital. Bar graphs were generated using GraphPad Prism 8.3 (GraphPad Software, San Diego, CA, USA).

## 3. Results

### 3.1. Optimization of Injection Time and Dose of Indocyanine Green for Lung Cancer Detection in a Preclinical Study

**Figure 1:**
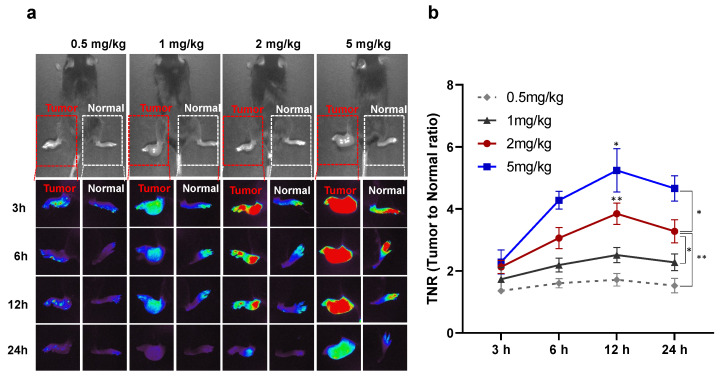
Time and dose optimization of ICG for tumor detection in mice model with footpad tumor. (**a**) NIR fluorescence imaging of footpad of mouse treated with ICG at different doses (0.5, 1, 2, and 5 mg/kg) and times (3, 6, 12, and 24 h). The normal and tumor footpad were detected by NIR fluorescent imaging system. (**b**) Quantification of NIR fluorescent signal of TNR in tumor of mouse model that received ICG at different doses and times. TNR in tumor tissues was quantified using Image J software. The red square dotted line indicates the tumor site and white square dotted line indicates the normal site. *, *p* < 0.05; **, *p* < 0.01.

We optimized the dosage and time of ICG injection for footpad tumor detection in a mouse tumor model. The mean volume of the footpad tumor of mouse was 72.1 ± 31.5 mm^3^ (41.2–144.5 mm^3^). As shown in Figure 1, ICG fluorescence signals could be detected in tumors injected with ICG at all doses and at all time points. It was shown that the higher the dose of ICG, the better the contrast between the tumor and normal tissue. There was a statistically significant interaction between TNR with the injection dose and time of ICG (*p* < 0.01). Nevertheless, in footpad tumors of mice injected with 0.5 and 1 mg/kg ICG, the TNR was low, and lung cancer could not be precisely detected by the NIR imaging system (0.5 mg/kg, 1.4 ± 0.1 and 1 mg/kg, 1.7 ± 0.4). Therefore, to accurately detect tumors, ICG should be injected at a dose of 2 mg/kg (TNR at 3 h, 1.4 ± 0.1; 6 h, 3.1 ± 0.3; 12 h, 3.8 ± 0.4; 24 h, 3.2 ± 0.4). Figure 1b shows that ICG was initially distributed throughout the body, regardless of whether it was a tumor or normal tissue after ICG injection, but it was washed from 6 h in normal tissue. At all doses, the ICG fluorescence signal was the highest at 12 h after injection (TNR at 0.5 mg/kg, 1.7 ± 0.2; 1 mg/kg, 2.4 ± 0.2; 2 mg/kg, 3.8 ± 0.4; 5 mg/kg, 5.2 ± 0.7). For the tumor injected with 2 mg/kg and 5 mg/kg, the fluorescence signal at 12 h was significantly higher than that at other time points. Thus, injecting 2 mg/kg ICG 12 h before surgery can minimize the dose to reduce ICG-related side effects and accurately detect tumors (Figure 1).

### 3.2. Intraoperative Fluorescence Image-Guided Thoracoscopic Resection of Lung Cancer in a Rabbit Model

**Figure 2:**
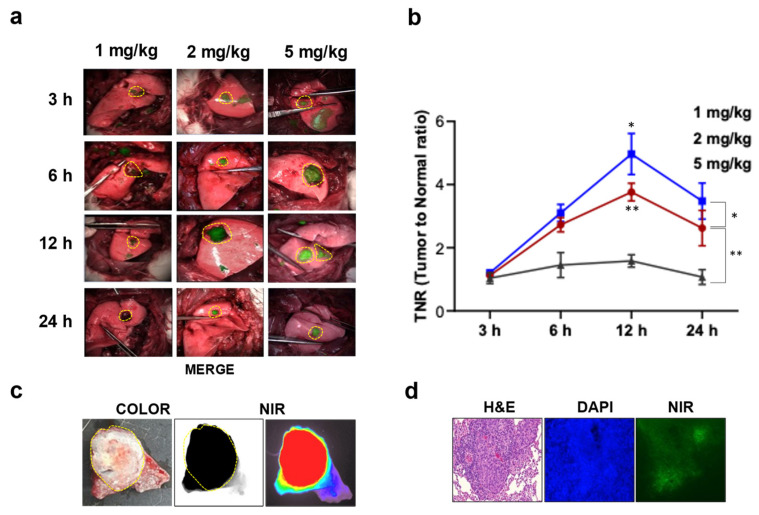
Time and dose optimization of ICG for lung cancer detection in rabbit model with lung cancer. (**a**) Representative merged image (NIR image merged with color image) of lung cancer in rabbit model treated with ICG at different doses (1, 2, and 5 mg/kg) and times (3, 6, 12, and 24 h). The lung cancer was imaged by near-infrared fluorescent imaging system. (**b**) Quantification of NIR fluorescent signal of TNR in lung cancer in rabbit model that received ICG at different doses and times. TNR in tumor tissues was quantified using Image J software. *, *p* < 0.05; **, *p* < 0.01. The yellow circular dotted line indicates the tumor site. (**c**) Representative ex vivo lung image of bright-field white light NIR in rabbit with lung cancer model after ICG intravenous injection. The lung cancer rabbit model was established 2–3 weeks after VX2 cells injection. (**d**) Representative image of histology, DAPI, NIR of lung cancer.

For preoperative imaging of lung tumors, CT/PET scans were performed on three randomly selected rabbits (Supplementary Figure S1). Lung cancer models were successfully established in all 33 rabbits confirmed by histopathological examinations. The dose and time optimization were performed in the rabbit lung cancer model. Consistent with the results obtained from the mouse cancer model, lung cancer could be accurately detected in rabbits injected with 2 (3.8 ± 0.2) and 5 mg/kg (5.0 ± 0.4) of ICG, and the TNR of these doses was highest at 12 h (*p* < 0.01 and *p* < 0.05, Figure 2). Based on the optimized study, we successfully detected lung cancer and performed lung cancer surgery using an endoscopic system in a rabbit lung cancer model (Supplementary Video S1 and Figure S2). We further examined the distribution of ICG in lung cancer using microscopic imaging. Ex vivo data showed that ICG was mainly distributed in the lung cancer area. Moreover, microscopic NIR fluorescence and histological images of lung cancer showed that ICG was dominantly distributed in lung cancer cells (Figure 2).

### 3.3. Identification of Lung Cancer Using Indocyanine Green Fluorescent Imaging in Patients with Lung Cancer

Based on the optimal ICG injection time and dose derived from the preclinical studies, we evaluated the efficacy of ICG for detecting lung cancer in 51 patients with NSCLC (Table 1). The patient sample comprised 32 men and 19 women, with a mean age of 65 ± 10 years (range, 38–81 years). The surgical methods were: wedge resection in 3 cases, segmentectomy in 5 cases, lobectomy in 42 cases, and pneumonectomy in 1 case. All procedures were successfully completed by VATS or robotic surgery, with no conversion to the open procedure. The mean cancer diameter was 2.8 ± 1.5 cm (range, 0.7–7 cm). The pathological diagnoses were adenocarcinoma in 31 cases (61%), squamous cell carcinoma in 16 cases (31%), sarcoma in two cases (4%), and neuroendocrine carcinoma in two cases (4%). The pathological TNM staging showed 14 cases (27%) for T1N0M0, 23 cases (44%) for T2N0M0, 6 cases (12%) for T3N0M0, 2 cases (4%) for T1N1M0, 4 cases (8%) for T2N1M0, and 1 case (2%) for T4N1M0 and T2N2M0, respectively. No intraoperative or postoperative adverse events related to the ICG injection were observed.

**Figure 3:**
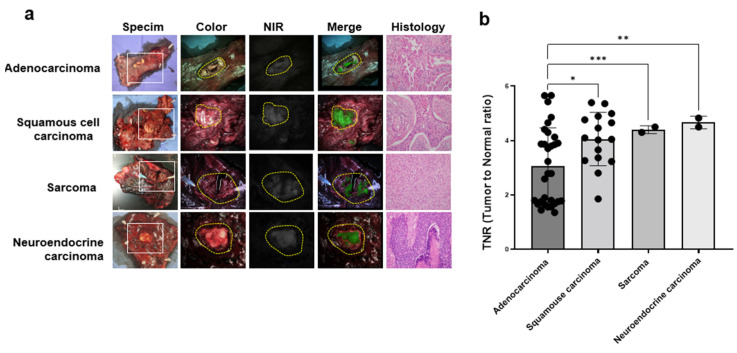
Identification of lung cancer using ICG fluorescent imaging in patients with lung cancer. (**a**) Representative images of preoperative CT, specimen, color, NIR, and merged (color and NIR) histology of lung cancer in patients treated with ICG (2 mg/kg) before 12 h surgery. (**b**) The quantification of NIR fluorescent signal of TNR in lung cancer with a different type of histology. The yellow circular dotted line indicates the tumor site. *, *p* < 0.05; **, *p* < 0.01; ***, *p* < 0.001.

We compared the TNR of ICG in patients with lung cancer. Figure 3 showed that the TNRs of squamous cell carcinoma (4.1 ± 1.0), sarcoma (4.4 ± 0.1), and neuroendocrine carcinoma (4.6 ± 0.2) were significantly higher than that of adenocarcinoma (3.0 ± 1.4). Among the 51 cases, the ICG fluorescent signal was successfully detected in 18 cases (58%) of adenocarcinoma, 15 cases (94%) of squamous cell carcinoma, two cases (100%) of sarcoma, and two cases (100%) of neuroendocrine carcinoma.

Among the 14 cases with no fluorescence signal, two pathologically confirmed cases (one case of adenocarcinoma and one case of squamous cell carcinoma) were a solid tumor with black pigments. The remaining 12 adenocarcinomas were confirmed to be pure or mixed GGN nodules by preoperative CT and pathology was confirmed as adenocarcinoma in situ (*n* = 2), lepidic (*n* = 5), acinar (*n* = 3), and micropapillary (*n* = 2) patterns, respectively.

### 3.4. Comparison of the Tumor-to-Normal Ratio in Lung Cancer Based on the Consolidation-to-Tumor Ratio

**Figure 4:**
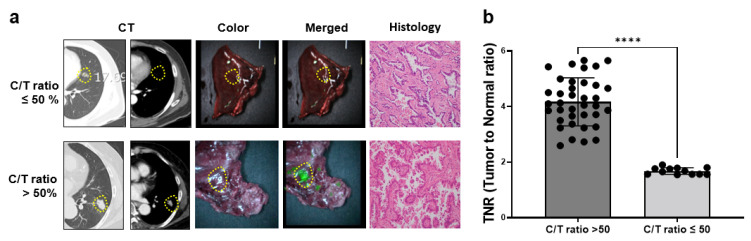
Comparison of ICG-based TNR of lung cancer according to C/T ratio by intravenous injection. (**a**) Representative image of preoperative CT, specimen, color, merged (color + NIR), and histology of lung cancer. (**b**) The quantification of NIR fluorescent signal of TNR in lung cancer with different C/T ratio. A yellow circular dotted line indicates the tumor site. ****, *p* < 0.0001.

To determine whether the NIR fluorescence signal of ICG is associated with the solid portion of lung cancer, we divided 30 patients with adenocarcinoma into two groups based on the C/T ratio: those with a C/T ratio ≤ 50% (12 cases) and those with the C/T ratio >50% (18 cases). We then compared the TNR with the C/T ratio > 50% group with solid tumors, which included 18 cases of adenocarcinoma, 15 cases of squamous cell carcinoma, two cases of sarcoma, and two cases of neuroendocrine carcinoma. Figure 4 shows that the NIR fluorescence image was clearly visible in the C/T ratio > 50% group but not in the C/T ratio ≤ 50% group. The TNR of the C/T ratio > 50% group (4.1 ± 0.9) was significantly higher than that of the C/T ratio ≤ 50% group (1.6 ± 0.1) (*p* < 0.001).

**Figure 5:**
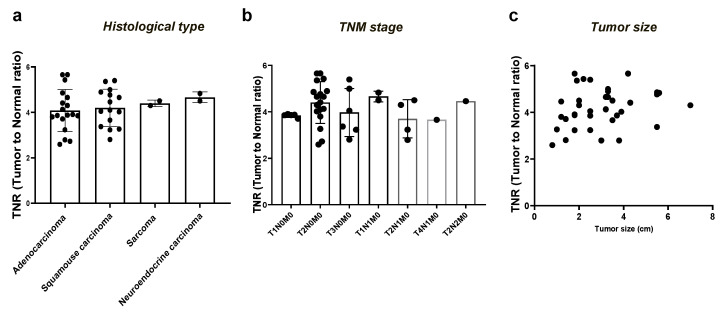
Assessment of NIR fluorescent signal of TNR in lung cancer according to histological type (**a**) TNM stage (**b**), and size of tumor (**c**) in lung cancer. NIR, near-infrared; TNR, tumor-to-normal ratio.

We analyzed the TNR in 37 patients with lung cancer and detected ICG fluorescent signals to assess whether the TNR was affected by the histological type, TNM stage, and size of lung cancer. As shown in Figure 5, there were no significant differences in the TNR of lung cancer according to histological type (*p* = 0.79) and TNM stage (*p* = 0.57). In addition, the TNR was not corelated with tumor size (r = 0.24, *p* = 0.16).

### 3.5. Evaluation of the Feasibility of ICG-Based Lung Cancer Detection in Clinical Practice

**Figure 6:**
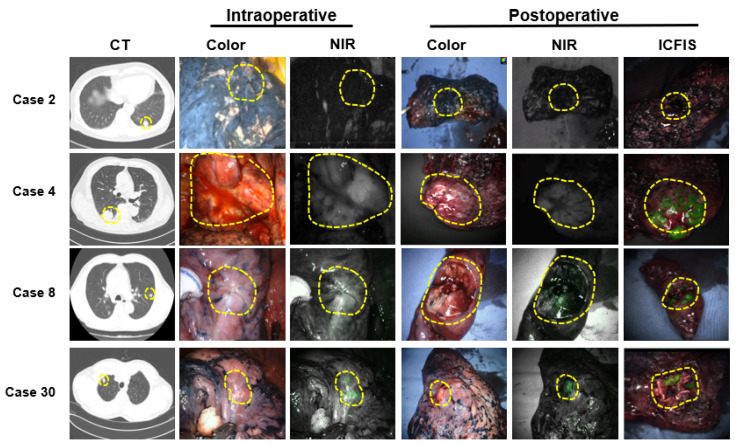
Evaluation of intraoperative and postoperative NIR fluorescence images of lung cancer using PinPoint (Case 2 and Case 4) or a robotic-assisted system (Case 8 and Case 30). A yellow circular dotted line indicates the tumor site.

We further evaluated the feasibility of an ICG injection protocol for lung cancer detection in 11 of the 51 patients using the fluorescence imaging system used in our hospital. Consistent with the results of ICFIS detection, it is difficult to detect lung cancer with anthracosis (case 2) or located at a depth of 0.5 cm (case 8) using intravenously injected ICG during surgery. However, for tumors at a depth of 0.5 cm, the fluorescent signal was detectable when the tumor was excised ex vivo to expose it. Superficial lung cancer was successfully detected either intraoperatively or postoperatively using a PinPoint (case 4) or robot-assisted system (case 30) (Figure 6 and Supplementary Video S2).

## 4. Discussion

Multiple studies have performed intravenous injection of ICG for lung cancer detection, including primary [26] and metastatic lung cancer [35]. Nevertheless, most of these studies were limited to solid tumors, and no study has examined lung GGNs, the incidence of which has steadily increased in recent years. In the present study, we explored the feasibility of ICG-based lung cancer imaging in 12 cases of adenocarcinoma presenting with GGNs and the C/T ratio ≤ 50%. Overall, 51 cases of lung cancers with a C/T ratio > 50% were successfully detected, with a detection rate of 95%. However, lung cancers with a C/T ratio ≤ 50% were not detected using ICG images. Therefore, this study can provide guidance regarding the use of ICG for lung cancer detection, especially for early-stage lung cancers.

For the detection of lung cancer, an injection of 5 mg/kg ICG 24 h before surgery is the commonly used method, based on the flank tumor model [30]. Nonetheless, based on our previous studies on thymoma and esophageal cancer, we know that different methods of ICG injection should be used to detect different types of tumors [23,24]. Therefore, dose and time optimization are required for lung cancer models rather than flank tumor models. In addition, although studies in small animal models for the clinical application of the ICG injection method are important, studies in medium-sized animal lung cancer models are particularly important for minimally invasive surgery [34].

In this study, dose optimization was performed at a low level (0.5, 1, 2, and 5 mg/kg) in a footpad tumor model of mice, in order to minimize the dose while accurately detecting tumors. In addition, the ICG injection method for lung cancer detection was optimized for the first time using a rabbit lung cancer model. From preclinical results, we found that when using ICG to detect cancer, higher doses resulted in a higher contrast between the tumor and normal tissues. At least 2 mg/kg or more should be injected to correctly detect lung cancer (Figure 1 and Figure 2). At all doses and in all animal models, the ICG fluorescence signal was the highest 12 h post-injection. Thus, we believe that injecting 2 mg/kg of ICG 12 h before surgery is a method that can minimize the dose to reduce ICG-related side effects and accurately detect lung cancers. This result is consistent with that of Jack et al. [30], who found that the TNR was highest 12–24 h after injection. The 2 mg/kg dose is lower than the 3 mg/kg recommended in a dose optimization study that included patients with lung cancer, conducted by Newton et al. [36]. In this study, the 1 mg/kg ICG dose was reduced because the intravenously injected ICG was gradually washed out not only in the normal tissues but also in the tumor over time. Thus, the tumor distribution of ICG at 12 h was higher than that at 24 h. We successfully performed lung cancer resection in the rabbit model based on a protocol optimized for rabbit cancer models. In addition, microscopic imaging of the lung cancer margin confirmed that ICG was distributed in the tumor cells. Moreover, the feasibility of using the optimized ICG injection method to detect lung cancer was evaluated in 51 patients with lung cancer. ICG detected 58% of adenocarcinomas, 94% of squamous carcinomas, and 100% of both neuroendocrine carcinomas and sarcomas.

We further evaluated the 14 cases without a fluorescence signal: two pathologically confirmed cases were solid tumor with black pigments, and the remaining 12 cases were confirmed as adenocarcinoma with pure or mixed GGN nodules by pathology and preoperative CT. From our results, we identified two barriers to lung cancer detection using ICG. First, anthracotic lung tissue was detected with a low TNR of lung cancer, which is consistent with the results of several other studies [37]. Anthracosis generates a black pigment through the deposition of carbonaceous particles that absorb large amounts of energy/light and increase the parenchymal fluorescence. This results in an elevated background parenchymal fluorescence signal, ultimately leading to a poor TNR [37]. Second, the ICG fluorescence signal was high in solid tumors, such as squamous cell carcinoma, whereas it was low in adenocarcinoma with a C/T ratio ≤ 50% in GGN nodules. Consequently, no difference was found upon comparing the TNR of adenocarcinoma with a C/T ratio >50% to that of other types of lung cancer. Furthermore, comparing the TNRs of 12 cases of the C/T ratio ≤ 50% group with the solid lung cancer group showed that the TNR of lung solid cancer was significantly higher than that of the C/T ratio ≤ 50% group. Hence, the low fluorescence signal in adenocarcinoma is due to GGN nodules with the C/T ratio ≤ 50%. Accordingly, our data suggested that ICG detection of lung cancer is dependent on the solid portion of lung cancer.

The accumulation of ICG in solid cancers is associated with vascular permeability at the cancer site. It is driven by passive targeting of tumor cells through the intrinsic membrane-binding capacity of ICG, the high endocytic activity of tumor cells, and disrupted tight junctions [38]. Pulmonary GGNs are becoming an important clinical dilemma as their diagnosis is increasing owing to the introduction of low-dose CT scans. GGN nodules were not detected by ICG because of the failure of the cells at normal and tumor demarcation to induce increased vascular permeability and the disruption of tight junctions. This resulted in insufficient ICG uptake. However, the reasons for this need to be clarified in future studies. Consequently, ICG is expected to be able to replace invasive marking methods in lung cancer with the C/T ratio of > 50%. Notwithstanding this, the development of contrast agents for targeted lung cancer with the C/T ratio of ≤ 50% is needed in future studies. OTL38 has been reported to detect GGN in lung cancer, and it may be the contrast agent that overcomes the limitations of ICG [39].

Lastly, we confirmed the feasibility of ICG-based detection of lung cancer during surgery using the imaging system commonly used in our hospital in 11 patients (Figure 5 and Supplementary Video S2). Consistent with ex vivo results of ICFIS detection, lung cancer was successfully detected either using a PinPoint or robot-assisted system (Figure 6 and Supplementary Video S2). Hence, we demonstrated that the ICG injection method can be directly applied in the surgical field.

Although we successfully detected lung cancer using ICG, this study has some limitations. First, even though the injection dose of ICG was reduced to 2 mg/kg in this study, owing to the low quantum dose (QY) of ICG (7.8), the injection dose still far exceeds the tumor-targeting dose currently under development with OTL38 (0.025 mg/kg) [40] or cRGD-ZW800-1 (0.005 mg/kg) [41]. Second, ICG-based NIR fluorescent image-guided surgery has depth limitations (5–10 mm). To overcome the depth problem associated with NIR-I-based ICG fluorescence images, contrast agents such as NIR-II [42,43,44] or NIR/PET [45,46], which can detect tumors deeper than 1 cm, are being developed. Accordingly, further development of NIR-II fluorescent imaging agents or hybrid multimodal contrast agents that can detect deep tumors, especially for GGN nodules, is needed to overcome the limitations of NIR-based ICG fluorescence images.

## 5. Conclusions

For intraoperative lung cancer detection, ICG at 2 mg/kg 12 h before surgery was the optimal injection method. Lung cancers with a C/T ratio > 50% were successfully detected using ICG with a detection rate of 95%, but not with a C/T ratio ≤ 50%. ICG is not a cancer-specific agent, and its low quantum yield leads to low TNR. Therefore, further research is needed to develop fluorescent agents targeting lung cancer, especially for GGN nodules.

## Figures and Tables

**Table 1:** Characteristics of patients with lung cancer.

Characteristics	*n* = 51
Mean age (Range)	65 ± 10 (38–81)
Gender (%)	
Female	19 (35%)
Male	32 (65%)
Tumor location (%)	
RUL	11 (22%)
RML	4 (8%)
RLL	12 (25%)
LUL	13 (25%)
LLL	11 (20%)
Surgical procedure (VATS or Robotic) (%)	
Wedge resection	3 (8%)
Segmentectomy	5 (8%)
Lobectomy	42 (82%)
Pneumonectomy	1 (2%)
Mean tumor size, cm (Range)	2.8 ± 1.5 (0.7–7)
Histologic diagnosis, (%)	
Adenocarcinoma	31 (61%)
Squamous cell carcinoma	16 (31%)
Sarcoma	2 (4%)
Neuroendocrine carcinoma	2 (4%)
p-TNM stage (%)	
T1N0M0	14 (27%)
T2N0M0	23 (45%)
T3N0M0	6 (12%)
T1N1M0	2 (4%)
T2N1M0	4 (8%)
T4N1M0	1 (2%)
T2N2M0	1 (2%)

## Data Availability

The data presented in this study are available upon request from the corresponding author.

## Supplementary Materials

The following supporting information can be downloaded at: https://www.mdpi.com/article/10.3390/cancers15143643/s1, Figure S1: Fluorescent image guided lung cancer surgery in rabbit model with lung cancer; Figure S2: ICG based intraoperative fluorescence image-guided thoracoscopic resection of lung cancer in rabbit model; Video S1: Intraoperative fluorescence image-guided thoracoscopic resection of lung cancer in rabbit model with lung cancer; Video S2. Intraoperative fluorescence image-guided thoracoscopic resection of lung cancer in lung cancer patient.
