# Supplementary material for: Optimization of Indocyanine Green for Intraoperative Fluorescent Image-Guided Localization of Lung Cancer; Analysis Based on Solid Component of Lung Nodule
Source: Cancers (Basel). 2023 Jul 16;15(14):3643. doi: 10.3390/cancers15143643 (PMC10377801; doi:10.3390/cancers15143643)
Supplement: Supplementary file 1 [file cancers-15-03643-s001.zip › cancers-2469671-supplementary figures.pdf]

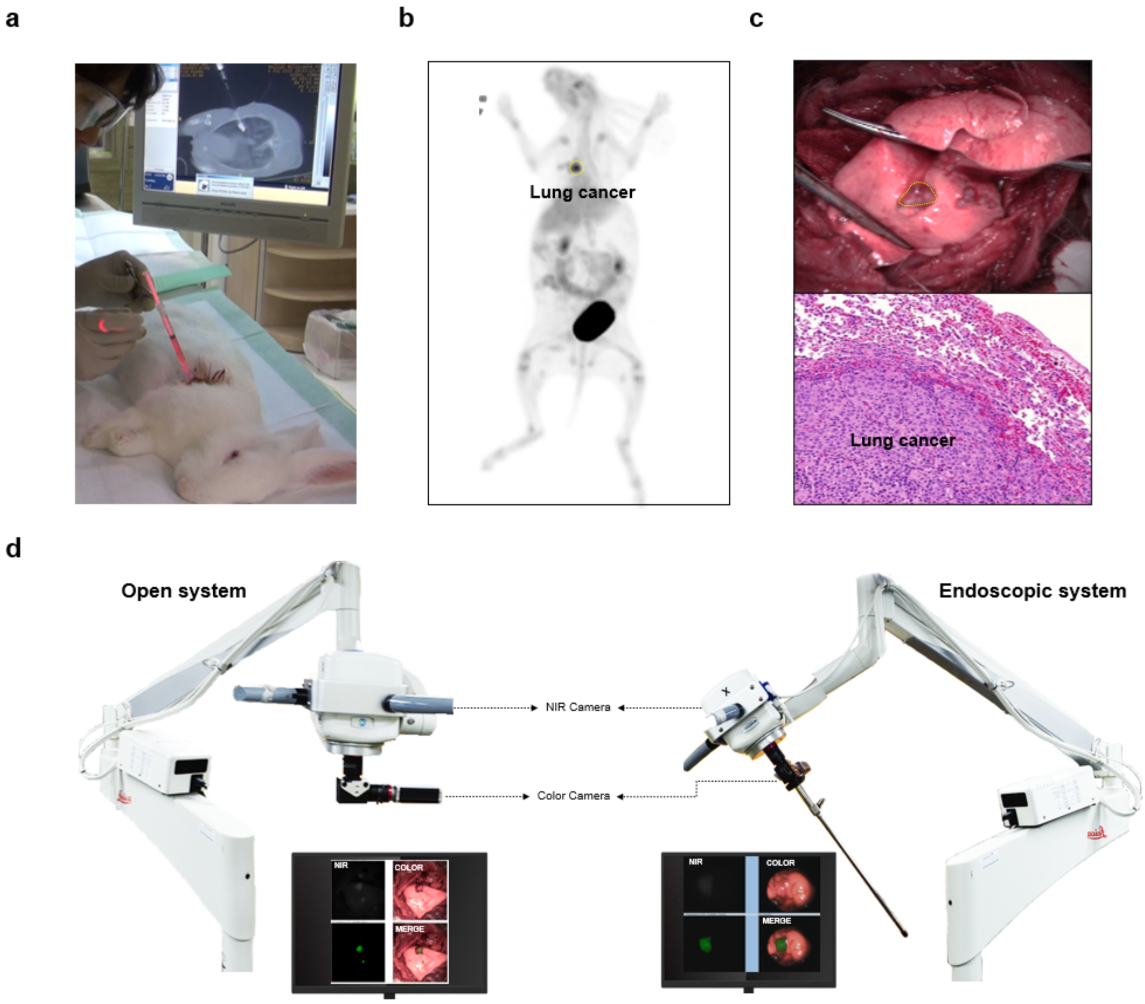

Figure S1. Fluorescent image guided lung cancer surgery in rabbit model with lung cancer. The rabbit lung cancer model was established by CT-guided direct injection of VX2 cells, and the establishment of the cancer model was confirmed by PET/CT and hematoxylin and eosin staining (a–c). (d) An overall view of intraoperative color and fluorescence imaging system (ICFIS) with open and endoscope system. CT, Computed tomography; NIR, near-infrared.

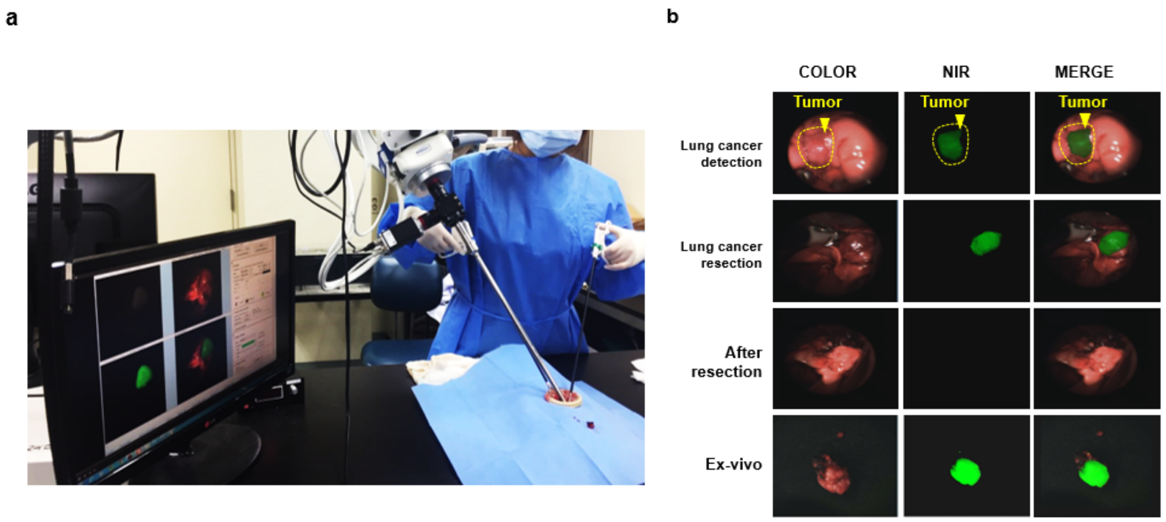

Figure S2. ICG based intraoperative fluorescence image-guided thoracoscopic resection of lung cancer in rabbit model. (a) Representative image of ICG guide lung cancer surgery using ICFIS endoscope system. (b) Representative image of color, NIR, and Merged (color+NIR) of lung cancer at pre and post-surgery. The yellow circular dotted line indicates the tumor site. NIR, near-infrared ; TNR, tumor to normal ratio.
